# Supplementary material for: A Pilot Study of Circulating Monocyte Subsets in Patients Treated with Stem Cell Transplantation for High-Risk Hematological Malignancies
Source: Medicina (Kaunas). 2020 Jan 18;56(1):36. doi: 10.3390/medicina56010036 (PMC7023283; doi:10.3390/medicina56010036)
Supplement: Supplementary file 1 [file medicina-56-00036-s001.pdf]

medicina

MDPI

SUPPLEMENTARY INFORMATION

A PILOT STUDY OF CIRCULATING MONOCYTE SUBSETS IN PATIENTS TREATED WITH STEM CELL TRANSPLANTATION FOR HIGH-RISK HEMATOLOGICAL MALIGNANCIES

Ida Marie Rundgren [1,2], Elisabeth Ersvær [1], Aymen Bushra Ahmed [3], Anita Ryningen [1] and Øystein Bruserud [2,3] *

[1] Department of Biomedical Laboratory Scientist Education and Chemical Engineering, Faculty of Engineering and Natural Sciences, Western Norway University of Applied Sciences, Bergen, Norway
[2] Department of Clinical Science, University of Bergen, Bergen, Norway
[3] Section for Hematology, Department of Medicine, Haukeland University Hospital, Bergen, Norway

Supplementary Table 1. Detailed clinical and biological characteristics of the patients included in the study. Conventional AML chemotherapy refers to the previously published European guidelines for handling of AML patients; the treatment was then in accordance with these guidelines [1]. The comorbidity score refers to the status at the pretransplant evaluation (patients 1-11) or the status before the chemotherapy cycle when the monocyte subset levels were tested.

Id	DIAGNOSIS AND GENETIC ABNORMALITIES	IMMUNOPHENOTYPING OF MALIGNANT CELLS	INITIAL PRETRANSPLANT TREATMENT AND RESPONSE	CMI [2]
**CONVENTIONAL INDUCTION AND CONSOLIDATION FOLLOWED BY STEM CELL TRANSPLANTATION**				
1	Acute lymphoblastic leukemia, L2 morphology<br>Genetic analysis: t(9;22) with bcr-abl translocation (b3a2), in addition t(10;14)	CD5$^-$CD10$^-$CD19$^+$CD20^{hetero}CD34$^+$CD39^{hetero}	Pretransplant induction and conditioning according to the NOPHO protocol for 11 weeks [3].	0
2	MDS-high risk with IPSS-R score 7 [4, 5]<br>Karyotype: del17(q10)	Not available	Complete remission with after conventional induction chemotherapy (daunorubicin+cytarabine).	0
3	PLL-T<br>Karyotyping not available.	Morphology and immunophenotype typical for T-PLL (details of the immunophenotype not available)	Complete hematological remission induction after alemtuzumab therapy for 9 weeks.	2
4	MDS-HR2 with 15 % blasts and IPSS score 4.5.<br>Karyotyping not available.	Not available	Complete hematological remission after one conventional induction cycle (daunorubicin+cytarabine).	1
5	AML secondary to MDS. Results from genetic testing are not available.	Not available	Complete hematological remission induction achieved after conventional induction chemotherapy (daunorubicin+cytarabine).	0
6	AML secondary to MDS with trisomy 11	Not available	Complete remission after two daunorubicin+cytarabine cycles.	0
7	AML de novo with normal karyotype<br>Flt3-ITD with ratio 0.41, no NPM1 abnormality	CD11b$^-$CD13$^+$CD14^{hetero}CD33$^+$CD34$^-$ CD45^{weak}CD64^{hetero}CD117^{hetero}HLA.DR$^+$	Remission after one induction cycle (daunorubicin+cytarabine), one consolidation cycle with intermediate cytarabine before allotransplantation.	0
8	AML de novo med inv(3;3)del(5); nor Flt3 or NPM1 abnormalities	CD13$^+$CD14$^-$CD33$^+$CD34$^+$CD117$^+$HLA-DR^{weak}	Blast reduction and normalized peripheral blood counts after conventional induction therapy.	2
9	AML de novo with inv(16)	CD11b$^+$CD113$^+$CD14$^-$CD33$^+$CD34$^-$CD117$^-$	Complete remission after one induction (daunorubicin+cytarabine) and one consolidation cycle.	0

10	Molecular relapse of APL with t(15;17). Normal karyotype and MRD negative pretransplant	Morphology and immunophenotyping typical for APL	Induction with conventional chemotherapy. MRD negative prior to transplantation [6, 7].	0
11	AML de novo, normal karyotype and no Flt3 orNPM1 mutations, biallelic CEBPA mutation.	CD7$^+$CD13$^+$CD14$^-$CD34$^+$CD45^{weak}CD117$^+$HLA-DR$^+$MPO$^+$	Complete molecular remission after first induction (daunorubicin+cytarabine) cycle, one consolidation cycle before autotransplantation.	0
**CONVENTIONAL INDUCTION AND/OR CONSOLIDATION CHEMOTHERAPY**				
12	AML de novo with t(8;21) No Flt3 or NPM1 abnormalities	CD11b$^-$CD13$^+$CD14$^-$CD33$^+$CD34^{hetero}CD117$^+$HLA-DR$^+$MPO$^+$	Conventional induction chemotherapy (anthracycline+cytarabine); complete remission after first induction. Tested during the two first consolidation cycles	0
13	AML relapse with normal karyotype and no Flt3 or NPM1 abnormalities	CD11b$^+$CD13$^+$CD14$^-$CD33$^+$CD34^{hetero}CD117$^+$HLA-DR$^+$MPO$^+$	Induction with etoposide, amsacrine and cytarabine; achieved complete hematological remission after one cycle	0
14	AML de novo with normal karyotype and no FLt3 or NPM1 abnormalities	CD11b$^-$CD13^{weak}CD14$^-$CD34$^+$CD45^{weak}CD117$^+$MPO$^+$. Positive for erythroid markers	Complete remission after first induction (daunorubicin+cytarabine); tested during the first consolidation cycle	0
15	AML de novo with del(9) and no FLt3 or NPM1 abnormalities	CD11b$^-$CD13^{weak}CD14$^-$ CD34$^+$CD38$^+$CD45^{weak}CD117$^+$CD117$^+$MPO$^+$	Induction treatment with daunorubicin plus cytarabine, no detectable blasts in the bone marrow at the time of testing	1
16	AML relapse Normal karyotype	CD11b$^-$CD13$^+$CD14$^-$CD34$^-$CD45^{weak}CD64$^-$CD117$^+$HLA-DR$^+$MPO$^+$	Induction: with daunorubicin plus cytarabine leading to remission. First consolidation cycle with mitoxantrone, cytarabine.	1

Abbreviations: ALL, acute lymphoblastic leukemia; AML, acute myeloid leukemia; APL, acute promyelocytic leukemia; F, female; Hetero, heterogeneous; M, male; MDS-HR2, myelodysplastic syndrome, high-risk class 2; MPO, myeloperoxidase; MRD, minimal residual disease; PLL-T prolymphocyte leukemia T cell type.

Supplementary Table 2. Monocyte and neutrophil reconstitution after conditioning therapy followed by stem cell transplantation. All 11 stem cell transplant recipients (autotransplant recipients marked with *) were included in this part of the study. The table presents the first posttransplant day with normal total monocyte cell counts (normal level 0.4-1.3 x 10^9/L) in peripheral blood, the monocyte level on this day together with the corresponding neutrophil level (lower normal limite 1.7 x 10^9/L) and the additional days from normalization of peripheral blood total monocyte counts until normalization of the neutrophil counts. The ***variation range*** is indicated together with the ***median*** for each of the four parameters,

**Patient identity**	**First posttransplant day with normalized monocyte levels**	**Monocyte level at the first day of normalization**	**Neutrophil levels at the first day of normal total monocytes**	**Additional days from the first day of normal monocytes until normalized neutrophil levels**
1	+11	***0.15***	0.3	12
2	+15	***0.05***	0.0	5
3	+13	0.04	0.1	4
4	***+18***	0.04	***0.2***	9
5	+13	0.07	0.2	8
6	+11	0.11	***0.0***	***17***
7	+11	0.20	0.0	8
8	**+9**	0.17	***0.6***	***7***
9*	+10	0.17	0.2	5
10*	***+11***	***0.49***	0.1	***3***
11*	+10	0.31	0.2	4

REFERENCES

1. Dohner, H:Estey, E, et al., *Diagnosis and management of AML in adults: 2017 ELN recommendations from an international expert panel.* Blood, 2017. **129**(4): p. 424-47 DOI: 10.1182/blood-2016-08-733196.
2. Sorror, ML:Giralt, S, et al., *Hematopoietic cell transplantation-specific comorbidity index as an outcome predictor for patients with acute myeloid leukemia in first remission: combined FHCRC and MDACC experiences.* Blood, 2007. **110**(13): p. 4606-13 DOI: 10.1182/blood-2007-06-096966.
3. Toft, N:Birgens, H, et al., *Results of NOPHO ALL2008 treatment for patients aged 1-45 years with acute lymphoblastic leukemia.* Leukemia, 2018. **32**(3): p. 606-15 DOI: 10.1038/leu.2017.265.
4. Garcia-Manero, G, *Myelodysplastic syndromes: 2015 Update on diagnosis, risk-stratification and management.* Am J Hematol, 2015. **90**(9): p. 832-41.
5. Steensma, DP, *Myelodysplastic syndromes current treatment algorithm 2018.* Blood Cancer J, 2018. **8**(5): p. 47 DOI: 10.1038/s41408-018-0085-4.
6. Yanada, M:Takami, A, et al., *Autologous hematopoietic cell transplantation for acute myeloid leukemia in adults: 25 years of experience in Japan.* Int J Hematol, 2019 DOI: 10.1007/s12185-019-02759-y.
7. Ganzel, C:Mathews, V, et al., *Autologous transplant remains the preferred therapy for relapsed APL in CR2.* Bone Marrow Transplant, 2016. **51**(9): p. 1180-3 DOI: 10.1038/bmt.2016.96.

© 2019 by the authors. Submitted for possible open access publication under the terms and conditions of the Creative Commons Attribution (CC BY) license (http://creativecommons.org/licenses/by/4.0/).
